# Comparison and Optimization: Research on the Structure of the PET Bottle Bottom Based on the Finite Element Method

**DOI:** 10.3390/polym14153174

**Published:** 2022-08-03

**Authors:** Shangjie Ge-Zhang, Mingbo Song, Zehang Huang, Maodan Li, Liqiang Mu

**Affiliations:** 1 College of Science, Northeast Forestry University, Harbin 150040, China; gzsj19@nefu.edu.cn; 2 College of Forestry, Northeast Forestry University, Harbin 150040, China; songmb@126.com; 3 Diotec Semiconductor Shanghai Co., Ltd., Shanghai 201500, China; zhh.2022@outlook.com; 4 Encare Instrument Technology (Shenzhen) Co., Ltd., Shenzhen 518100, China; lmd.2000@outlook.com

**Keywords:** computer modeling, carbonated drinks, beverage packaging, stress cracking, simulations

## Abstract

The polyethylene terephthalate (PET) beverage bottle is one of the most common beverage packages in the world, but the bottom of the PET bottle tends to crack due to excessive stress. In this paper, through numerical simulation and finite element analysis, the mechanical properties of four typical geometric models of bottle bottom are studied, and it is determined that “claw flap bottle bottom (CF-bottom)” has the best structure. Then, the shapes of four bottle bottom structures are fine-tuned by using the automatic optimization method. Under the premise of the same material quality, the surface maximum principal stress, the overall maximum principal stress, and the total elastic strain energy of the bottle bottom are reduced by 46.39–71.81%, 38.16–71.50%, and 38.56–61.38%, respectively, while the deformation displacement is also reduced by 0.63 mm–3.43 mm. In contrast to other papers, this paper dispenses with the manual adjustment of various variables, instead adopting automatic shape optimization to obtain a more accurate model. The percentage of maximum principal stress reduction is remarkable, which provides a feasible theoretical guidance for the structural optimization of PET bottle bottom in the production process.

## 1. Introduction

Polyethylene terephthalate (PET) is a linear thermoplastic polyester with light weight, high strength, dimensional stability, and other properties. It has been used in many industrial fields [1,2,3], especially in the packaging of bottled beverages, and the consumption in this sector has been increasing in recent years [4]. Due to the limitations of early blow molding technology, in order to evenly distribute the pressure the earliest bottled drinks were designed to be hemispherical at the bottom, with detachable base parts to keep the bottle stable. With the development of molding technology, the structure of bottle bottoms has been diversified and integrated in production. The prefabricated PET beverage bottle with “body and base” has been replaced, and the most famous succedaneum is the design of the “claw flap (CF)” bottle bottom structure [5]. These integrated designs reduce the production cost of beverage bottles, simplify the production steps, and are more convenient and environmentally friendly. However, due to structural changes, the stress concentration of beverage bottles is more obvious, and the bottom of the bottles is more likely to be broken or cracked, resulting in liquid leakage [6].

At present, the stress research on PET bottles mainly focuses on the injection molding process and preparation materials. Cho et al. [7] reduced the maximum residual stress and shrinkage of PET beverage bottles by 22% and 25%, respectively, by adjusting the relationship between the parameters such as temperature, pressure, and injection time during injection-blow molding. Wang et al. [8] simulated the stretch blow molding process by finite element method (FEM) using a viscoplastic material model, and revealed the influence of temperature and other factors on thickness distribution and the deformation process. In addition, the orientation and crystallinity of PET molecules are important factors that determine the quality of the mechanical properties of the finished products [9,10,11,12]. Tracey et al. [13] studied the molecular morphology of the bottom of PET bottles by small angle X-ray scattering (SAXS), and found that the over-filling of preforms during injection, or circumferential stretching during blowing, were the reasons for the decrease of mechanical strength caused by crystallinity and molecular orientation. However, most studies are about the characteristics of PET materials themselves, without considering the geometric structure of the products [14,15,16]. In fact, the structural design of the bottle bottom is one of the important factors that affect the maximum pressure inside the PET bottle [17,18].

This work explored the optimal structure of the PET beverage bottle bottom, which effectively alleviated the fracture phenomenon of the PET bottle bottom caused by stress concentration. Firstly, a variety of PET beverage bottles were collected, and bottle bottom parameters were recorded, which were summarized into four types of typical bottle bottom structure models. Finite element simulation analysis was conducted to compare the maximum principal stress of the four types of typical bottle bottom models, and the CF-bottom structure was determined to be the best structure. Secondly, the shape of CF-bottom structure was optimized to determine the optimal geometry and total elastic strain energy of the structure. This work provides the theoretical basis for strengthening the mechanical structure of the PET bottle bottom, and could provide guidance for the production of molds, which has definite practical significance.

Considering that automatic optimization can only change the set parameters such as thickness and depth in a small range, but can not change the fixed parameters such as the number of claws, in order to optimize the bottle bottom structure to a greater extent, the further research direction of this subject is to carry out preliminary structure optimization by controlling variables, and then carry out automatic optimization. Finally, according to the results, the production process is adjusted to solve the practical problems encountered in the process of industrial blow-molding of PET bottles.

## 2. Principle and Experiment

### 2.1. Computer-Aided Modeling of Bottle Bottom Structure

Several common PET beverages in China were purchased, and four typical structural models were selected after induction, as shown in Table 1. In SolidWorks simulation software, the first three bottle bottom models were equally divided into five identical 72° segments, and the octagonal radial bottle bottom was equally divided into eight 45° segments. The total bottom was generated by duplication and rotation of this slice with rotating array component.

**Figure 1:**
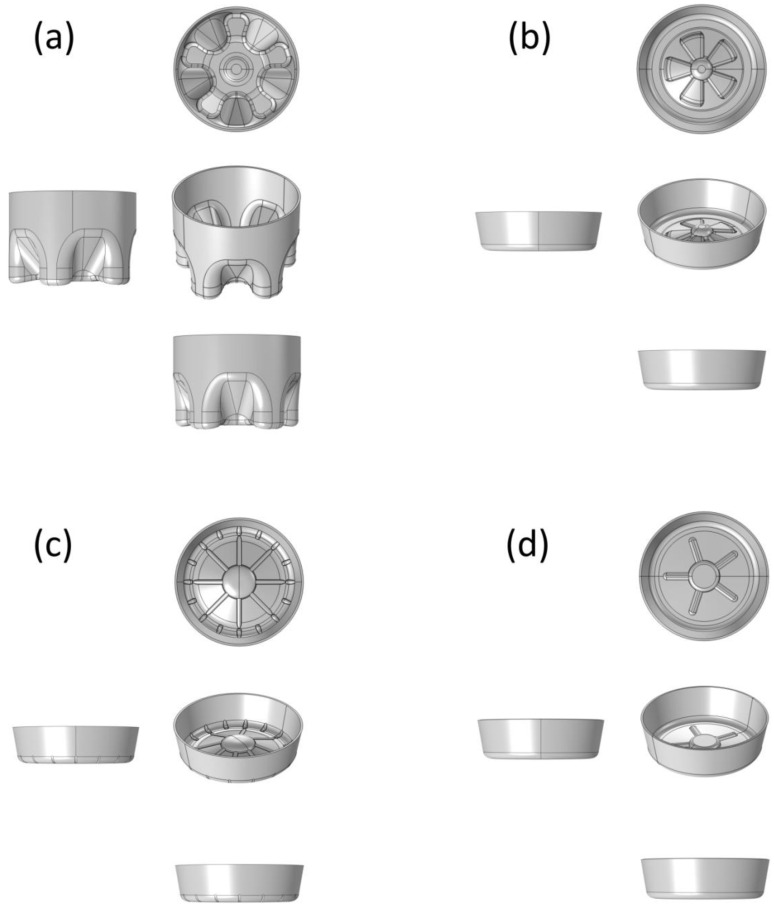
Diagram of CF-bottom (**a**), PP-bottom (**b**), PR-bottom (**c**) and OR-bottom (**d**) structure models.

In order to compare only the performance of the bottle bottom structure model, other influences such as environment and time during blow molding were not considered. In this paper, a uniform thickness of 1 mm was assumed first. Figure 1 is the diagram of four typical bottle bottom structure models simulated by SolidWorks.

### 2.2. Selections of Material and Finite Element Mesh

The core material of the bottom was made of PET plastic 1 mm in thickness. The density of the material is taken as 1190 kg m^-3^. The values of Young’s modulus, Poisson’s ratio, and thermal expansion coefficient for the material are taken as 3200 MPa, 0.35, and 7 × 10^−5^ K^−1^, respectively. The specific characteristics and test indexes of PET are given in Table 2.

The results of finite element analysis depend on the shape and size of meshes, but high-quality meshes are not equal to the most detailed meshes. Too-small meshes will waste computing resources, and the processing time will increase with the increase of meshes, which may also lead to non-physical solutions. Triangular mesh division was adopted for the bottom model with complex geometry, which took into account the excellent boundary adaptability of triangular mesh, prevented the generation of bad elements, and made the calculation converge quickly. Polygonal elements with more sides had higher accuracy than triangular elements, so the mesh number of triangular elements would be appropriately increased to make up for the lack of accuracy. Due to the difference of bottle bottom structure, the size of mesh unit and the number of meshes are different (Table 3).

### 2.3. Pressure Setting

Comsol was used to carry out finite element simulation analysis. A pressure of 0.4 MPa (0.04 kg mm^−2^) was applied to the bottom of the bottle, which was the pressure value in the bottle when the carbon dioxide gas content used in the stress cracking test is 20 °C. The force direction was outward along the normals of the bottom. The Von Mises stress distributions of different models under the same load were obtained.

### 2.4. Constraint Conditions

Before stress testing and shape optimization, some necessary constraints need to be added to make the results conform to reality, and the optimized model can be applied to product production. Sudden sharp protrusions or depressions are not allowed by actual production.

#### 2.4.1. Fixed Edge

The fixed edges of the constraint were set as the inner and outer wall edge of the upper edge, which prevented translations and rotations of the rigid body. The upper edges were chosen because the research goal was the bottom of the bottle, not the body, and the fixed edge was an extension of the bottle bottom, which is linked to the bottle body. We directly set the constraint Equation (1) of fixed edge:

(1) d=0

Before optimization, the fixed edges were added with fixed constraint and prescribed displacement.

#### 2.4.2. Free Shape Domain

The nonlinear Yeoh smoothing type [19] was used to determine the deformation of the mesh in each domain. Compared with other methods [20,21,22,23], Yeoh smoothing prevented the further deformation of these regions to some extent, and effectively distributed the mesh deformation in the domain more evenly, away from the moving boundary. The strain energy expression is as follows:

(2) $$W=C_{1}\left(I_{1}-3\right)+C_{2}{\left(I_{1}-3\right)}^{2}+C_{3}{\left(I_{1}-3\right)}^{3}$$

The value of stiffening factor $C_{2}$ in the equation controls the nonlinear hardening of artificial materials under deformation, and was set to 10 in the experiment; $I_{1}$ is Cauchy-Green deformation tensor.

#### 2.4.3. Free Shape Boundary

A free shape boundary was added to the edge of the free shape domain, and the points on the boundary can move in the area of $\pm d_{max}$. In order to make the calculation result converge, $d_{max}$ with a value of 1 mm was the set maximum allowable displacement. The constraint Equation (3) of free shape boundary is as follows:

(3) $$d=c+R_{min}^{2}\nabla_{\|}^{2}d,\;\;\;\;\;\;-d_{max}\leq c_{i}\leq d_{max}$$

where $d_{max}$ is the maximum displacement and $R_{min}$ is the filtering radius.

### 2.5. Steady State Solver

Considering that the matrix of the model is sparse, that is, 0 accounts for more than 95% of the matrix, the steady-state solution adopts the PARDISO direct solver based on LU decomposition, which is located in the Intel Mathematical Kernel Library (MKL). The direct solver uses more memory than the iterative solver, but it is more robust.

### 2.6. Optimizations of Models

The thickness and the material distribution of the bottle bottom can significantly affect its mechanical properties [24]. In the optimization, the maximum principal stress and total elastic strain energy of the bottle bottom were mainly considered under the condition of using the same quality materials. Stress is an important factor determining whether the bottle bottom cracks. When the stress on the bottom of the bottle exceeds its maximum value, the bottom will crack. Stress concentration also makes the bottom of the bottle more prone to crack [25]. When a bottle with high internal pressure is opened, the elastic strain energy stored in the deformed bottle bottom will be released, giving energy to the liquid in the bottle, and the work of the liquid will shake and overflow from the bottle mouth. Excellent structure and material distribution should effectively reduce the elastic strain energy.

Automatic shape optimization was adopted to optimize the bottle bottom, which changed the geometric parameters, thickness, and material distribution parameters of the model. The solver method is the method of moving asymptotes (MMA) [26], which is a three-level nonlinear algorithm based on gradient. The simplest MMA is to generate an approximation subproblem in which the original function is replaced by a convex function in each iteration, based on the gradient information and moving asymptote of the current iteration point. After being solved, the only optimal solution will become a new subproblem at the next iteration point. But what is used here is the globally convergent version of MMA (GCMMA) [27], which includes external iteration and internal iteration. Each external iteration may require one or more internal iterations, or it may not require internal iteration. The subproblems of the *k*-th internal iteration under the *v*-th external iteration are as follows:

(4) $$\mathrm{minimize}\;{\tilde{f_{0}}}^{\left(k,v\right)}\left(x\right)+z+\frac{1}{2}d_{0}z^{2}+\overset{m}{\underset{i=1}{\sum }}\left(c_{i}y_{i}+\frac{1}{2}y_{i}^{2}\right)$$

(5) $$\mathrm{subject}\;\mathrm{to}{\tilde{f_{i}}}^{\left(k,v\right)}\left(x\right)-a_{i}z-y_{i},\;\;\;i=1,\ldots ,m$$

(6) $$\alpha_{j}^{\left(k\right)}\leq x_{j}\leq \beta_{j}^{\left(k\right)},\;\;\;\;\;\;\;\;\;\;\;\;\;\;\;\;\;\;\;\;\;\;\;\;\;j=1,\ldots ,n$$

(7) $$y_{i}\geq 0,\;\;\;\;\;\;\;\;\;\;\;\;\;\;\;\;\;\;\;\;\;\;\;\;\;\;\;\;\;\;\;\;\;\;\;\;\;\;\;\;\;i=1,\ldots ,m$$

(8) z≥0

The approximate functions are constructed as:

(9) $${\tilde{f_{0}}}^{\left(k,v\right)}\left(x\right)=\overset{n}{\underset{j=1}{\sum }}\left(\frac{p_{ij}^{\left(k,v\right)}}{u_{j}^{\left(k\right)}-x_{j}}+\frac{q_{ij}^{\left(k,v\right)}}{x_{j}-l_{j}^{\left(k\right)}}\right)+r_{i}^{\left(k,v\right)},\;i=0,1,\ldots ,m.$$

Between each outer iteration, the bounds $\alpha_{j}^{\left(k\right)}$ and $\beta_{j}^{\left(k\right)}$ and the asymptotes $l_{j}^{\left(k\right)}$ and $u_{j}^{\left(k\right)}$ are updated. The maximum iteration interval of optimization in this work was 10–20, and its purpose was to obtain the optimal structure corresponding to each model on the premise of convergence.

## 3. Results and Discussion

### 3.1. Results of Computer-Aided Modeling

As mentioned above, in the process of model comparison, we only compared the influence of the geometric structure of the model on the mechanical properties of the bottle bottom, but did not consider the thickness change and thickness distribution for the time being, so the thickness value was defined as 1 mm. It is worth noting that the bottom of the bottle is not a regular shape due to its convex or concave structure. In SolidWorks modeling, if the thickness of all irregular shapes was defined as 1 mm, cracks or cliffs would appear at the convex–concave interface. With the shell extraction command, all parts were smoothly connected without cliffs, and the average value of the final result was still 1 mm (Figure 1).

### 3.2. Results of von Mises Stress Surface Distribution

**Figure 2:**
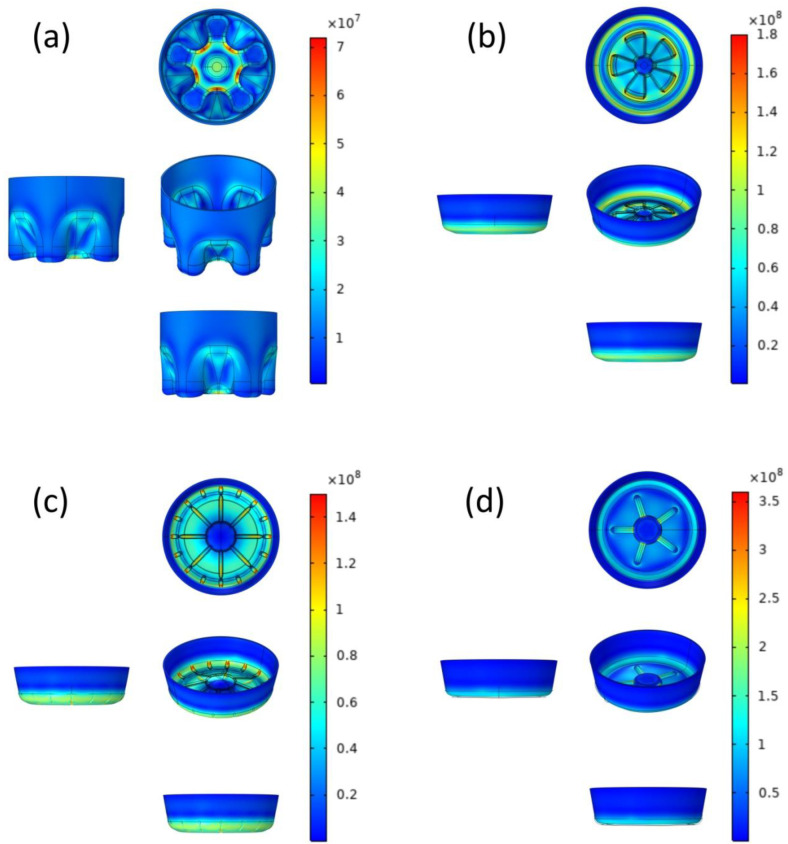
The Von Mises stress (Pa) surface distribution of pre-optimized models CF-bottom (**a**), PP-bottom (**b**), PR-bottom (**c**), and OR-bottom (**d**).

The Von Mises stress surface distribution at the bottom of bottle before optimization is shown in Figure 2, showing the average thickness of 1 mm with four types of effective stress distribution of the bottle. The thickness standard value was the average thickness of the bottom area, although the bottle thickness was unevenly distributed, but as the first step in the contrast, we only compared the model of the structure’s influence on the mechanical properties of the bottle. As can be seen from Figure 2a, the maximum stress of the CF-bottom model is 7.13 × 10^7^ Pa, and the minimum value is 1.6 × 10^6^ Pa; the maximum principal stress is distributed in the outer ring of the center of the bottom of the bottle, showing a state of outward divergence, and there is an obvious stress concentration phenomenon at the bottom of the groove. The maximum stress of the PP-bottom model is 1.76 × 10^8^ Pa, while the minimum is 8.49 × 10^5^ Pa; the maximum principal stress is distributed in the outer arc of the petal, especially at the apex (Figure 2b). The stress range of PR-bottom model in Figure 2c is from 1.07 × 10^6^ Pa to 3.6 × 10^8^ Pa; the stress concentration is obvious at the joint between the inner ring of the bottle bottom and the groove, and the outer ring of the center of the bottle bottom diverges outwards, which is the location of the maximum principal stress distribution. The OR-bottom model’s maximum stress and minimum stress are 1.49 × 10^8^ Pa and 2.09 × 10^5^ Pa, respectively; the maximum principal stress shown in Figure 2d is distributed in the lateral edge far from the center, and the lateral depression is the most obvious. To sum up, the circumferential crack formed by computer simulation of bottle bottom cracking should be divided into two typical types. One is that the outer ring cracks circumferentially, and the cracked part is located around the outer ring at the bottom of the bottle, especially at the junction of the outer ring and the groove, that is, the end of the groove. Another typical crack type is the circumferential crack of the inner ring, which is located around the center of the bottom of the bottle, and mainly at the joint between the center and the groove. This is consistent with the actual cracking test results [28,29]. The circumferential crack is caused by the bottom geometry and stress concentration. Most of the cracks that lead to leakage appear in the bottom of the bottle, where the material is not stretched enough and its strength is weak [30,31]. In fact, the crack at the bottom of the bottle is not only caused by insufficient tensile strength, but is also related to the structural design of the bottom of the bottle [32,33,34].

Among the four, the maximum surface stress of CF-bottom under the same load is less than that of other bottle bottom structures, which is a better geometric structure model. The distribution of the maximum principal stress is basically consistent with the cracking phenomenon in the surface stress cracking test, which proves that the maximum principal stress plays a key role in the cracking process [35,36]. In addition, it should be pointed out that the maximum stress of the material sometimes appears inside the object, and at this time, the maximum stress of the surface is not the maximum stress of the whole, which is mentioned in later sections.

### 3.3. Optimization Results

#### 3.3.1. Reduction of Overall von Mises Stress and Total Elastic Strain Energy

**Figure 3:**
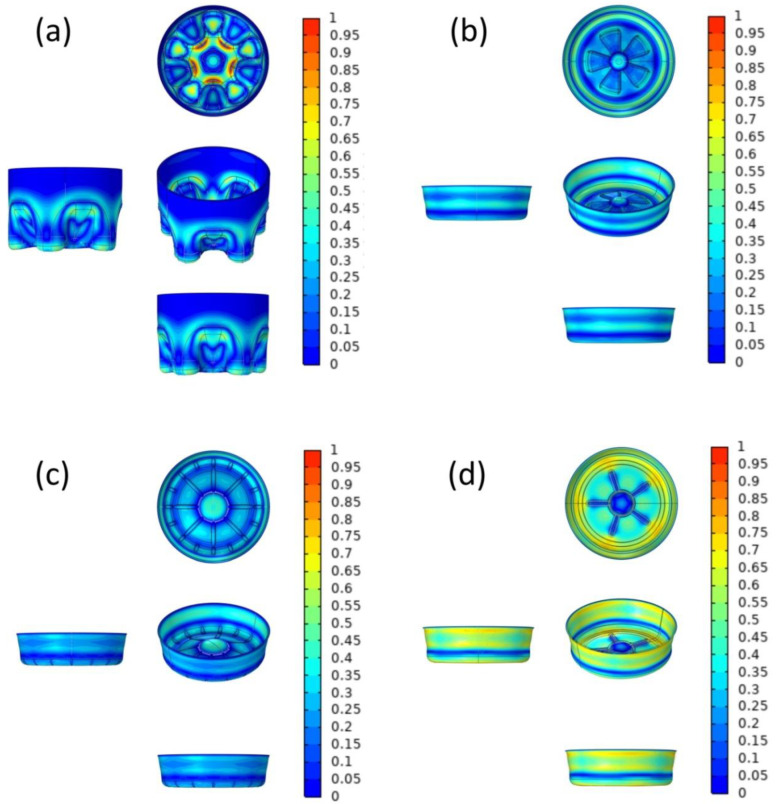
Comparison of model structures CF-bottom (**a**), PP-bottom (**b**), PR-bottom (**c**), and OR-bottom (**d**) before and after optimization.

Theoretically, the more materials are used in production, the greater the average thickness, the better the mechanical properties of the bottle bottom, but this was not meaningful for practical production. Considering the cost and other factors related to production, it was feasible to set up the optimization using the same material. Figure 3 demonstrates the comparison of the model structure before and after optimization. The range in the legend is the boundary displacement relative to the normal direction, that is, the movement of the material layout. The lowest value of 0 is no movement, and the highest value is 1 mm movement. Accordingly, compared with the original model, the overall maximum principal stresses of the four optimization models were reduced by 5.57 × 10^7^–2.00 × 10^8^ Pa, and the reduction rate ranged from 38.16% to 71.50% (Table 4). Actually, except CF-bottom model, the minimum stress of the other three models increases after improvement; this is due to the fact that the quality of consumed materials is unchanged, the materials in the area with less stress are misappropriated, and the thickness of the area is reduced, but the bottom fracture is caused by the maximum stress instead of the minimum stress.

**Figure 4:**
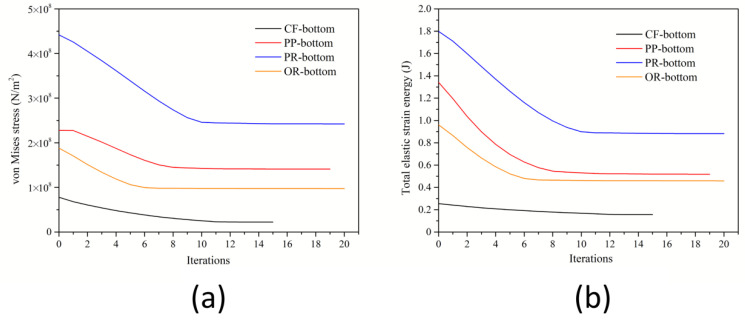
Diagrams of the overall maximum principal stress-iteration times (**a**) and the total elastic strain energy-iteration times (**b**).

In this paper, it is considered that the model has converged when the relative tolerance of the iterative solver is less than 10^−3^. Figure 4 shows the total maximum principal stress-iteration number diagram, and the total elastic strain energy-iteration number diagram of four types of bottle bottoms, from which it can be seen that the final qualitative image of Von Mises stress after optimization remains unchanged. It is worth noting that the overall Von Mises stress shown in Figure 4 is indeed higher than the surface Von Mises stress corresponding to Figure 2, because the maximum stress is located inside the bottle bottom instead of on the surface; however, the stress relationship among the four models still corresponds. In addition to the maximum principal stress causing the bottle bottom to break, we also consider the total elastic strain energy of the bottle bottom. This is because when the cap is opened, the gas in the bottle escapes, resulting in a sudden drop in pressure, and the bottom of the bottle springs back. The elastic strain energy contained in the bottle is transferred to the liquid in the bottle, and the liquid would overflow by shock. Excessive elastic strain will lead to more liquid spillage, resulting in an uncomfortable drinking experience. It can be seen directly that the elastic strain energy is obviously reduced by 38.56% to 61.38% after optimization. The reduction ratio of the PP-bottom is the smallest, which is due to its excellent structure before optimization.

#### 3.3.2. Comparison of Material Deformation Results

**Figure 5:**
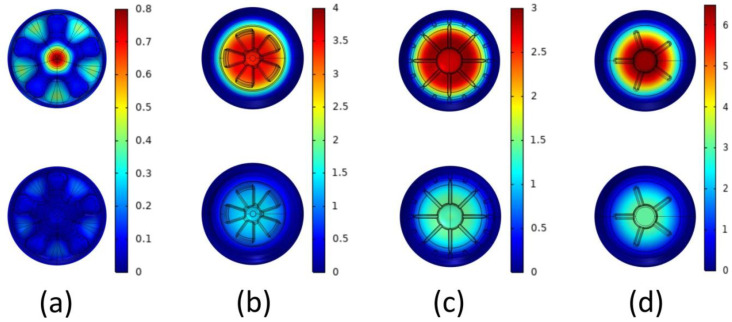
The deformation diagram of CF-bottom (**a**), PP-bottom (**b**), PR-bottom (**c**), and OR-bottom (**d**) before and after optimization (mm).

When other conditions remained the same and the pressure in the bottle was increased, the bottom of the bottle would be deformed. If the external force was excessive, deformation beyond the strength of the material itself, the material would fracture. Therefore, the size of deformation was also an important evaluation index [37]. We applied 0.4 MPa (0.04 kg mm^−2^) pressure to the model before and after optimization. The obtained material deformation results are shown in Figure 5. It can be clearly seen that the material deformation mainly occurs in the center of the bottom of the bottle, diverges outward and gradually decreases. Before and after optimization, the maximum deformation of the CF-bottom model is reduced by 0.63 mm, with a reduction rate of 77.78%. The maximum deformation of the PP-bottom model, PR-bottom model and OR-bottom model are reduced by 2.46 mm (60.74%), 3.43 mm (52.45%) and 1.51 mm (51.19%), respectively.

## 4. Conclusions

This paper mainly analyzed the influence of geometric structure on the bottom fracturing of PET bottles, comparing and optimizing four models. The cracks in the bottom of the bottle are caused by weak material strength and concentrated principal stress in the circumferential groove. Most of the cracks are circumferentially distributed in the inner or outer ring of the bottom of the bottle, especially in the groove-inner and outer ring junction where the maximum principal stress is located. The thickness, structure, material distribution, and other parameters of the bottle bottom will significantly affect its mechanical properties. It is cumbersome to manually change each factor one by one, and it may easily cause conflicts among variables, so the results cannot be optimized. Automatic shape optimization removes the limitations of manual control and allows for the comprehensive consideration of multiple factors under the condition of using the materials of the same quality, with more accurate results. The total elastic strain energy was also considered, which is prepared for the user experience at the time of sale. The overall maximum principal stress and total elastic strain energy of the optimized bottle bottom decreased by 71.50% (CF-bottom) and 61.38% (PP-bottom) at most, and the displacement caused by deformation decreased by 0.63 mm–3.43 mm. In addition to improving the existing bottle bottom geometry structure, the influence of material properties such as tensile ratio and crystallinity on bottle bottom fracture should also be explored, which will be the next focus of this project.

## Figures and Tables

**Table 1:** Names of beverages and corresponding shape models.

Names of Beverages	Names of Models
Cola (carbonated beverage)	Claw flap bottle bottom (CF-bottom)
Iced tea (black tea beverage)	Pentagonal petal bottle bottom (PP-bottom)
Pure water (pure water)	Pentagonal radial bottle bottom (PR-bottom)
Natural Water (natural water)	Octagonal radial bottle bottom (OR-bottom)

**Table 2:** Characteristics and test indexes of PET.

Characteristics	Value
Density	1190 kg m^−3^
Young’s Modulus	3200 MPa
Poisson’s Ratio	0.35
Thermal Expansion Coefficient	7 × 10^−5^ K^−1^
Intrinsic Viscosity	0.83 dL g^−^^1^
Minimum Burst Strength Requirement	7 bar

**Table 3:** The number of meshes for the models.

Names of Models	Number of Meshes
CF-bottom	30,137
PP-bottom	22,248
PR-bottom	126,324
OR-bottom	88,726

**Table 4:** Comparison of the overall maximum stress of the models.

Names of Models	Before Optimization Pa	After Optimization Pa	Decrement %
CF-bottom	7.79 × 10^7^	2.22 × 10^7^	71.50%
PP-bottom	2.28 × 10^8^	1.41 × 10^8^	38.16%
PR-bottom	4.42 × 10^8^	2.42 × 10^8^	45.25%
OR-bottom	1.88 × 10^8^	9.74 × 10^7^	48.19%

## Data Availability

Not applicable.
